# Clinical characteristic, management, and outcomes of cervical spinal brucellosis: a retrospective cohort study over 1-year postoperative follow-up

**DOI:** 10.1186/s13018-024-04868-9

**Published:** 2024-06-27

**Authors:** Tao Zhang, Lihua Ma, Hua Liu, Lian Zhang, Chengwei Yang, Songkai Li

**Affiliations:** 1 Department of Spine Surgery, The 940th Hospital of Joint Logistics Support Force of Chinese PLA, Lanzhou, Gansu China; 2 The First Hospital of Lanzhou University, Lanzhou, Gansu China

**Keywords:** Cervical brucellosis, Brucellar spondylitis, Epidural abscess, Debridement, Antimicrobial treatment

## Abstract

**Background:**

The incidence of cervical spinal brucellosis is low, only a few case reports have been published, and case series are not widely reported in the medical literature. Therefore, clinical features, management, and outcomes of cervical spinal brucellosis are relatively unknown. In this series, the authors report 15 cases of patients with cervical spinal brucellosis, including clinical characteristic, imaging findings, management plans, the institution’s experience, and outcomes at 1 year postoperatively.

**Methods:**

The study reviewed the clinical and radiographic records of 15 patients who received antimicrobial pharmacotherapy, and anterior cervical debridement and fusion for cervical spinal brucellosis. The data collected included patient demographic characteristics, spinal level affected, abscess, neurology, pathological reports, duration and type of antimicrobial regimens, details of orthopedic management, and complications incurred during the procedure.

**Results:**

Neck pain (100%) and limb paralysis (86.7%) were the most common clinical presentations, and the disease had a rapid progression. The C6-7 segment was the most commonly affected segment, followed by C4-5 and C5-6. Imaging commonly revealed epidural or paravertebral abscesses (80%). There was a significant improvement in the VAS, JOA, and NDI scores three months after surgery, and the scores continued to improve until the final follow-up. There was a statistically significant difference between the pre- and postoperative scores (*P* < 0.05). The ESR and CRP levels returned to normal within three months postoperatively, being 7.7 ± 4.5 mm/h and 7.55 ± 3.48 mg/L, respectively. There were statistically significant differences between the pre- and postoperative levels (*P* < 0.05). The positive rate of bacterial culture testing of pus or lesion tissues was only 40%, but blood cultures revealed an even lower positivity rate (33.3%). The average antimicrobial pharmacotherapy regimen duration was 6.1 ± 1.9 months. All patients achieved intervertebral bone fusion within 8 months (4.8 ± 1.4 months) after surgery and were cured with non-recurrence.

**Conclusions:**

Spinal brucellosis rarely affects the cervical region, but its impact is more dangerous due to potential complications such as paraplegia or tetraplegia arising from epidural abscesses that compress the spinal cord. Surgical debridement, along with essential antimicrobial therapy, is an effective strategy and can lead to satisfactory prognosis in managing cervical spinal brucellosis.

## Background

Brucellosis is a leading cause of spondylodiscitis and spondylitis in endemic areas. Brucellar spondylitis, the most common and severe osteoarticular manifestation of human brucellosis, may affect multiple structures, including the vertebral bodies, intervertebral discs, and paraspinal structures. Spinal involvement occurs in 2-60% of cases [1, 2], with the lumbar spine being the most commonly affected segment, followed by the thoracic and cervical segments [2–4].

Although spinal involvement with brucellosis occurs infrequently in the cervical area (4.3-9.7% in the literature) [2, 5, 6], it can lead to severe complications such as paraplegia or tetraplegia due to epidural abscesses with spinal cord compression. Due to the low incidence of cervical spinal brucellosis, only a few case reports have been published [7–9], and no case series are widely reported in the medical literature. Although the cervical vertebrae are rarely affected by brucellosis, this involvement is particularly dangerous due to potentially life-threatening complications such as paraplegia or tetraplegia caused by frequent epidural abscesses that lead to spinal cord compression [10, 11]. Early diagnosis and prompt treatment are crucial in the prevention of severe neurological complications associated with cervical spinal brucellosis.

In this study, we systematically reviewed and summarized a series of cases of brucellar spondylitis, which included 15 cases of cervical spinal brucellosis. We evaluated the demographic and clinical characteristics, antimicrobial treatment, and surgical outcomes of these patients and provided a summary of our experience managing and following up with patients diagnosed with cervical spinal brucellosis.

## Methods

### Aim

To summarize the clinical characteristics, imaging features, management plans, and outcomes at 1 year postoperatively of cervical spinal brucellosis.

### Study design

This retrospective cohort study was carried out using a consecutive patient pool with cervical spinal brucellosis, who presented to our department between January 2013 and December 2021. The diagnosis of cervical spinal brucellosis was confirmed through clinical assessments and serological analysis of patient’s blood using a standard tube agglutination test (STAT) for Brucella species with a minimum titer of 1:100 (fourfold titer). Additional diagnostic confirmation was carried out by isolating Brucella spp. from blood, tissue or other body fluids [12, 13]. Diagnostic criteria: (1) epidemiological exposure history; (2) clinical and imaging findings related to spondylitis, excluding other suspected infections; (3) Brucella culture positive or STAT positive (fourfold).

### Patient selection

**Fig. 1:**
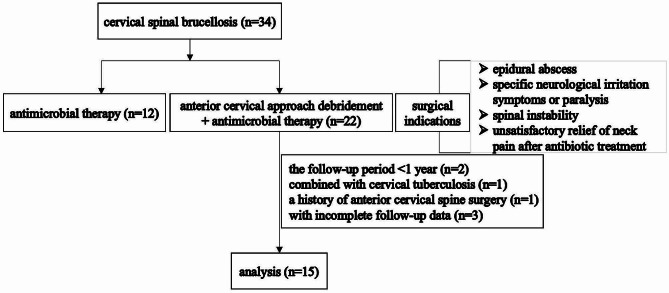
Flowchart for screening patients

Patients with a definite diagnosis of cervical spinal brucellosis who presented at least one of following were enrolled for surgical treatment: (1) clear imaging signs of epidural abscess; (2) specific neurological irritation symptoms or paralysis; (3) spinal instability; (4) unsatisfactory relief of neck pain after antibiotic treatment. The inclusion criteria were as follows: (1) a diagnosis of cervical spinal brucellosis; (2) treatment with anterior cervical approach debridement; and (3) at least 12 months of follow-up data. The exclusion criteria were: (1) concurrent infections such as cervical suppurative spondylitis or cervical tuberculosis; (2) a history of anterior cervical spine surgery; and (3) incomplete or unavailable follow-up data (Fig. 1).

### Patient management

All patients underwent a series of serum tests, including erythrocyte sedimentation rate (ESR), C-reactive protein (CRP), Rose Bengal plate agglutination test (RBPT), STAT, and imaging examinations via plain radiography, computed tomography (CT), and magnetic resonance imaging (MRI). Patients with fever also had blood cultures obtained. A standard antimicrobial pharmacotherapy regimen of doxycycline (200 mg/day) and rifampicin (600 mg/day) was administered to each confirmed patient, with additional combination therapy of ceftriaxone (2 g/day) for the first 3 weeks in patients with fever. Of the 15 patients studied, only 2 received preoperative antimicrobial agents, while the remaining 13 underwent timely surgery due to neurological deficits or severe and unbearable neck pain, without prior administration of preoperative antibiotics.

All patients underwent thorough anterior cervical debridement, abscess drainage, decompression, and spinal canal decompression as thoroughly as possible if space-occupying lesions were present. A suitable autologous iliac strut bone graft or interbody fusion cage filled with autologous bone was inserted into the intervertebral space. The surgical area was rinsed with copious amounts of saline until clear, and rifamycin (0.5 g) was added. Following surgery, resected lesions and pus were sent for pathological examination and bacterial culture. Nutritional enhancement was administered regularly during the perioperative period.

ESR, CRP, RBPT, and STAT levels were measured to evaluate the efficacy of antimicrobial treatments. After discharge, patients were examined monthly to assess their clinical responses and neurological function, including visual analog scale (VAS), Japanese Orthopedic Association (JOA), and neck disability index (NDI) assessments, ESR and CRP measurements; and to detect drug-related side effects and reactivation of the disease in its early stages. Radiography and CT were conducted every 3–6 months to evaluate bony fusion. The criteria for discontinuing antibiotics were as follows [13, 14]: (1) relief of spinal pain with disappearance of the inflammatory pattern; (2) normal body temperature; (3) normal CRP and ESR levels coupled with negative RBPT and STAT test results; and (4) changes in signal intensity on MRI.

### Statistical analysis

Statistical calculations were performed using SPSS version 20.0 (IBM Corp., Armonk, NY, USA). Continuous variables are presented as the mean ± standard deviation. A paired-samples *t* test was deployed to analyze the ESR, CRP, VAS, JOA, and NDI results, involving preoperative measurements, 3-month postoperative recordings, and final follow-up data. Statistical significance was indicated by a *P* < 0.05.

## Results

### Clinical characteristic

The study involved 15 participants, consisting of 9 males and 6 females, with a mean age of 55.5 ± 8.4 years (range 42–76 years) at presentation. The most common presenting symptom was neck pain (100%), followed by constitutional symptoms, such as night sweats, weight loss, malaise, and subjective limb weakness. Conversely, fever was not prevalent (40%). Spinal tenderness (73.3%) and paralysis (86.7%) were the most common clinical signs. Comprehensive details of the clinical signs and symptoms of the patients, as well as neurological assessments are provided in Table 1. All patients had epidemiological risk factors for exposure to microorganisms (Table 2). Of the cases, all had clinical and imaging manifestations of spondylodiscitis, only one had a multi-segment lesion, while the others had single-segment lesions. The lesions were located at different segments, particularly C3-4 (1 case), C4-5 (5 cases), C5-6 (4 cases), and C6-7 (6 cases).

**Table 1:** Clinical presentation of the patients in our series

Clinical symptoms and signs	No. of patients
**Symptoms**	
Neck pain Radicular pain Fever Subjective weakness of limbs Other constitutional symptoms (night sweats, weight loss, malaise)	15 7 6 9 10
**Clinical signs**	
Spinal tenderness Paralysis of limbs Loss of sensations Pyramidal-tract involvement Hyporeflexia/Hyperreflexia	11 13 8 8 4

**Table 2:** Clinical data and perioperative outcomes

Case No.	Age(years)/gender	Smoking/ Alcoholism /Drug use	Comorbidity	Risk factors	Course(weeks)	Segments	Abscess
1	59/F	N	N	Animal husbandry	8	C6-7	EA + PVA
2	53/M	Smoking	N	Animal husbandry	8	C6-7	EA + PVA
3	46/M	N	N	Animal husbandry	20	C5-6	EA
4	51/F	N	N	Animal husbandry	4	C6-7	PVA
5	50/M	N	HT	Animal husbandry	4	C4-5/C6-7	EA + PVA
6	57/M	N	HT	Animal husbandry	3	C4-5	N
7	52/M	N	N	Consumption of unpasteurized milk	4	C4-5	EA + PVA
8	66/M	N	DM	Animal husbandry	4	C4-5	N
9	54 M	Smoking	N	Animal husbandry	8	C5-6	PVA
10	76/M	N	HT	Veterinary worker	2	C6-7	EA + PVA
11	59/F	N	DM + HT	Consumption of unpasteurized milk	20	C3-4	N
12	49/M	Smoking	N	Animal husbandry	3	C5-6	EA + PVA
13	60 F	N	HT	Animal husbandry	2	C6-7	EA + PVA
14	58/F	N	N	Animal husbandry	36	C5-6	EA + PVA
15	42/F	N	N	Animal husbandry	4	C4-5	EA + PVA
Mean ± SD	55.5 ± 8.4				8.7 ± 9.5		

*M* male; *F* female; *N* no; *EA* epidural abscess; *PVA* paravertebral abscess; *DM* diabetes mellitus; *HT* hypertension

### Imaging features

**Fig. 2:**
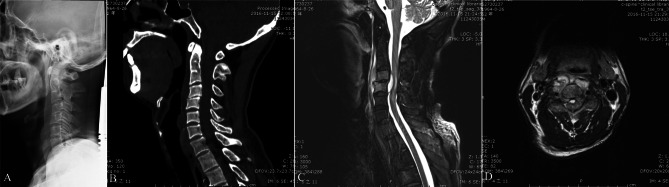
(**A**) Lateral radiograph shows loss of C4-5 intervertebral disc height. (**B**) Sagittal computed tomography image shows no sign of bone destruction. (**C**, **D**) Magnetic resonance imaging scans show the formation of paravertebral and epidural abscesses and spinal cord compression at the C4-5 level

Among the participants, 80% (12/15) had either a cervical epidural or paravertebral abscess, while one patient had a simple epidural abscess, and two had a simple paravertebral abscess. The most common was an epidural abscess with a paravertebral abscess (60%, 9/15) (Table 2; Fig. 2). Epidural and paravertebral abscesses in the cervical segment are usually connected via the intervertebral foramen. Although an epidural or paravertebral abscess is a common imaging feature of cervical spinal brucellosis, bone destruction is rarely observed on CT (Fig. 2).

### Perioperative outcomes

All patients received surgical treatment for neurological deficits or severe and unrelieved neck pain following a course of antimicrobial regimens. Table 2 summarizes the detailed perioperative outcomes of the patients.

### Postoperative outcomes

Preoperatively, the VAS, JOA, and NDI scores significantly improved three months after surgery and continued to improve until the final follow-up in all cases. The differences between the preoperative, 3-month postoperative, and final follow-up scores were statistically significant (*P* < 0.05, Table 3). Thirteen patients with preoperative limb paralysis showed significant neurological recovery in the JOA score post-surgery. However, the function of two patients had yet to fully recover to normal. Inflammatory symptoms disappeared at the final follow-up.

**Table 3:** VAS, JOA, and NDI score

	VAS	JOA	NDI
Pre-operation	5.7 ± 1.6	12.7 ± 3.7	25 ± 8.5
3 months post-operation	1.1 ± 0.9	15.9 ± 1.4	4.7 ± 1.7
Final follow-up	0.4 ± 0.5	16.4 ± 1.1	3.7 ± 1.2
*P*	< 0.001*/0.001**	< 0.001*/0.006**	< 0.001*/0.003**

*Compared with preoperative; ** Compared with 3 months postoperative

Before surgery, the average ESR and CRP levels were 35.5 ± 20.6 mm/h and 58.56 ± 44.42 mg/L, respectively, returning to normal within three months postoperatively. Statistically significant differences existed between the preoperative, 3-month postoperative, and final follow-up values (*P* < 0.05, Table 4). At three months after surgery and with antimicrobial regimens, the positive rate of RBPT remained high; however, STAT showed a significant decline. The RBPT and STAT test results were negative at the final follow-up (Table 4).

**Table 4:** Laboratory findings

	ESR (mm/h)	CRP (mg/L)	WBC	RBPT		STAT					
				Positive	Negative	Negative	1:50	1:100	1:200	1:400	1:800
Pre-operation	35.5 ± 20.6	58.56 ± 44.42	6.18 ± 2.70	15	0	0	0	2	8	4	1
3 months post-operation	7.7 ± 4.5	7.55 ± 3.48	5.76 ± 1.39	11	4	4	2	8	1	0	0
Final follow-up	6.7 ± 2.9	5.57 ± 1.56	6.03 ± 1.42	0	15	15	0	0	0	0	0
*P*	< 0.001*/0.373**	< 0.001*/0.009**	0.285*/0.301**	-	-	-	-	-	-	-	-

*Compared with preoperative; ** Compared with 3 months postoperative

**Fig. 3:**
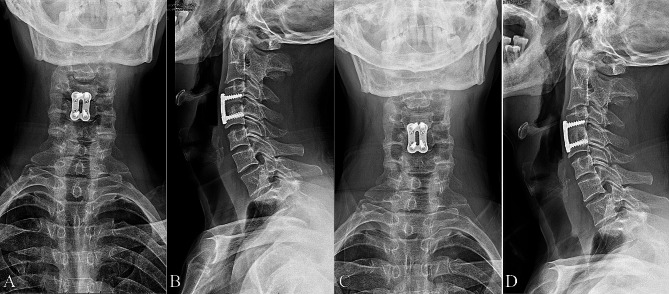
(**A**, **B**) Four months after surgery, the C4 and C5 interbody bone grafts had fused. (**C**, **D**) Twenty-seven months after surgery, the C4 and C5 interbody bone grafts achieved complete fusion and satisfactory cervical curvature

All cases underwent bacterial culture testing of pus or lesion tissues, but only 6 out of 15 samples tested positive (40%). Blood cultures were obtained only from febrile patients, revealing an even lower positivity rate of 2 out of 6 (33.3%) (Table 5). The bacterial types of the patients with positive bacterial cultures were Brucella melitensis. Histological analysis showed lymphocyte and monocyte infiltration, while acid-fast staining was negative. The patients were followed up for an average of 17.9 ± 5.2 months, and the average drug treatment duration was 6.1 ± 1.9 months. All patients achieved satisfactory intervertebral fusion, with an average bone fusion time of 4.8 ± 1.4 months (Fig. 3).

**Table 5:** Postoperative situation

Case no.	Pyo-culture	Blood culture	Pathology	Antimicrobial regimens	Duration of antimicrobial therapy (months)	Duration of follow-up (months)	Fusion time (months)	Complications	Outcomes
1	-	NA	Lymphocyte and monocyte infiltration, acid-fast staining (-)	DR	6	25	3	N	Complete recovery
2	+	+	Lymphocyte and monocyte infiltration, acid-fast staining (-)	C + DR	9	18	6	N	Complete recovery
3	-	-	Lymphocyte and monocyte infiltration, acid-fast staining (-)	C + DR	8	27	4	N	Complete recovery
4	-	NA	Lymphocyte and monocyte infiltration, acid-fast staining (-)	DR	6	15	6	N	Complete recovery
5	+	-	Lymphocyte and monocyte infiltration, acid-fast staining (-)	C + DR	5	15	5	N	Complete recovery
6	-	NA	Lymphocyte and monocyte infiltration, acid-fast staining (-)	DR	5	12	4	N	Residual bladder dysfunction
7	+	NA	Lymphocyte and monocyte infiltration, acid-fast staining (-)	DR	4	27	4	N	Complete recovery
8	-	NA	Lymphocyte and monocyte infiltration, acid-fast staining (-)	DR	9	20	6	N	Complete recovery
9	-	-	Lymphocyte and monocyte infiltration, acid-fast staining (-)	C + DR	9	22	8	N	Complete recovery
10	-	NA	Neutrophil and eosinophils infiltration, acid-fast staining (-)	DR	6	12	5	N	Complete recovery
11	-	NA	Lymphocyte and monocyte infiltration, acid-fast staining (-)	DR	4	15	4	N	Complete recovery
12	+	NA	Lymphocyte and monocyte infiltration, acid-fast staining (-)	DR	7	18	6	Y	Residual distal left upper limb muscle strength level 3
13	+	-	Lymphocyte and monocyte infiltration, acid-fast staining (-)	C + DR	4	14	4	N	Complete recovery
14	-	NA	Lymphocyte and monocyte infiltration, acid-fast staining (-)	DR	4	12	3	N	Complete recovery
15	+	+	Lymphocyte and monocyte infiltration, acid-fast staining (-)	C + DR	6	16	4	N	Complete recovery
Mean ± SD					6.1 ± 1.9	17.9 ± 5.2	4.8 ± 1.4		

*NA* not applicable; *N* no; *Y* yes

*DR* doxycycline 200 mg/day combined with rifampicin 600 mg/day

*C* + *DR* ceftriaxone 2 g/day, doxycycline 200 mg/day combined with rifampicin 600 mg/day

### Complications and prognoses

One patient experienced perioperative complications related to decompression, leading to residual limb dysfunction. Another patient suffered residual bladder dysfunction despite successfully curing cervical spinal brucellosis. Nevertheless, all other patients were cured, and their neurological functions returned to normal. None of the patients developed wound infection or infectious meningitis, nor was there a recurrence of cervical spinal brucellosis.

### Illustrative case

**Fig. 4:**
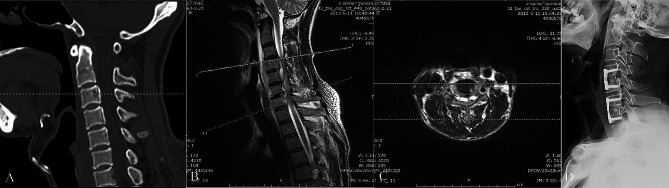
(**A**) Sagittal computed tomography image shows no sign of bone destruction. (**B**, **C**) Magnetic resonance imaging scans show the formation of paravertebral and epidural abscesses and spinal cord compression at the C4-7 levels. (**D**) Fifteen months after surgery, the C4-5 and C6-7 intervertebral bone grafts achieved complete fusion

A 50-year-old man presented with moderate neck pain for 4 weeks and progressive extremity weakness for 1 day. Preoperative cervical radiographs and CT scans did not indicate vertebral bone destruction. However, MRI revealed the formation of paravertebral and epidural abscesses, leading to spinal cord compression at the C4-7 levels (Fig. 4). The patient underwent an urgent anterior cervical debridement and spinal cord decompression. Three months post-surgery, the patient’s neurological function improved from grade B to grade D. During the follow-up period, cervical spinal brucellosis resolved (Fig. 4).

## Discussion

Brucellar spondylitis has a discernible regional distribution and is commonly found in the pasturing areas of northern China [15]. Meta-analysis has shown that majority of patients (43.7–79.4%) are employed in animal husbandry with a history of close contact with sheep and cattle, while a significant proportion of patients have a history of consuming uncooked meat or unpasteurized dairy products (11.5–78.8%). Additionally, 8.2% of the cases were associated with veterinary care [15, 16]. In this study, it was noted that cervical spinal brucellosis is mainly caused by direct or indirect contact with infected animals or the consumption of animal products, which is consistent with the literature available on the subject. The primary occupational risk identified in this study was animal husbandry, accounting for 80%, followed by the consumption of unpasteurized milk products (2/15).

Brucellar spondylitis is most common in males aged 45–54 years, with a mean age of 52.4 ± 16.6 years [5, 6, 17]. In this study, all 15 patients with cervical spinal brucellosis were over 42 years old, and the average age was 55.5 ± 8.4 years, consistent with previous reports [5]. The population most affected by cervical spinal brucellosis has demographic features in endemic areas where most patients are male (9/15), live in rural areas and have occupational risks. The susceptible segment for cervical spinal brucellosis is not clear, as no serial cases have been reported to date. Only a few case reports have identified susceptible segments located at the C4-5, C5-6, and C6-7 segments [7, 8, 10, 11, 18–20]. In this study, we found that the C6-7 segment was the most commonly affected segment, followed by C4-5 and C5-6. Detecting lesions in the C6-7 segment using radiography is challenging due to shoulder obstruction. Moreover, because cervical spinal brucellosis progresses rapidly with little bone destruction, this usually leads to missed abnormalities in the early stages of the disease.

Brucellar spondylitis symptoms are nonspecific, including spinal complaints, fever, fatigue, and sweating. The subtle nature of these symptoms and the lack of systemic signs, such as temperature elevation, often result in delayed diagnosis. Back pain (97-100%) is a common symptom of spinal brucellosis, and the fever incidence is between 55 and 61.5% [13, 21]. In our study, although neck pain (100%) was the most common symptom, the incidence of fever in patients with cervical spinal brucellosis was only 40%, which is lower than literature estimates. Therefore, in patients with short-term, severe neck pain living in endemic areas or high-risk groups, cervical spinal brucellosis should be considered as a differential diagnosis by physicians after a detailed clinical examination of the spine. Early recognition of complicated cases is critical to prevent devastating complications.

MRI findings indicate that 61.3% of brucellar spondylitis patients have abscesses, either epidural abscess (almost all cervical and thoracic regions) or paravertebral abscess [13, 22]. In this study, up to 80% of patients with cervical spinal brucellosis had abscess formation, with epidural abscess combined with paravertebral abscess being the most prevalent (60%). The incidence of brucellar spondylitis with epidural abscesses in the cervical segment is notably higher than that in the lumbar segment. Epidural and paravertebral abscesses in the cervical segment are usually connected via the intervertebral foramen, which can cause radiculopathy. Epidural masses may accompany the entire pathological process whereby compression of the nerve root or spinal cord may occur. When potential exposure to the infection is evident, an epidural abscess is identified, and cervical spinal brucellosis should be suspected. Therefore, MRI is the most reliable confirmatory test and the preferred imaging method for the diagnosis and follow-up of cervical spinal brucellosis and abscesses. Epidural abscesses may require immediate surgical intervention to prevent neurological deterioration.

The subtle presentation of brucellar spondylitis often causes diagnostic delays, but cervical involvement can lead to severe manifestations due to frequent epidural masses with spinal cord compression and neurological disabilities [7, 11]. Therefore, early diagnosis and prompt treatment are essential for preventing permanent neurological and spinal deformities. Although isolation of the organism from cultures is the gold standard for the diagnosis of brucellosis, positive cases range from 15 to 70% [23]. Our study found that only 33.3% (2/6) of blood cultures and 40% (6/15) of pyocultures tested positive. Biopsy isolation of Brucella is often challenging due to the intracellular localization of the bacterium, the chronic nature of the disease, and antimicrobial administration. Therefore, a diagnosis of brucellar spondylitis can be established presumptively by serology. The results showing an anti-Brucella titer ≥ 1:100 on standard tube agglutination tests indicate positive outcomes.

Antimicrobial therapy plays a critical role in treating brucellar spondylitis, with common drugs being streptomycin, rifampicin, doxycycline, and ciprofloxacin. The World Health Organization (WHO) recommends doxycycline (100 mg twice a day), rifampin (600 mg/day), and streptomycin (1 g/day, 21 days) for six months, which demonstrated higher efficacy rates [1, 24, 25]. However, doxycycline and rifampin combination therapy has been associated with relapses [24], while long-term antibiotics (usually over a three-month period) have proven effective in reducing relapses. Thus, because streptomycin was unavailable, our study administered a combination of doxycycline and rifampicin to all patients, while also providing concomitant ceftriaxone treatment for fever for three weeks. Complicated spinal brucellosis is defined as infection extension through the paravertebral and epidural spaces, psoas muscle, or radicles, regardless of neurological symptoms [16]. Ulu-Kilic et al. found in their study that successful therapy for complicated spinal brucellosis lasted for 16 weeks [16]. Our series treated all complicated cases with two or three antibiotics, with a mean treatment period of 6.1 ± 1.9 months. All patients made full recoveries without any relapses. To prevent relapse, we recommend at least six months of therapy until serology becomes negative.

In addition to essential antimicrobial agent therapy, surgical decompression is a final solution for cervical spinal brucellosis treatment when neurological deficits are caused by spinal cord or nerve root compression. Infrequent cases of progressive vertebral collapse or spinal instability with continuing systemic signs, despite adequate antimicrobial therapy, may also require surgery [14, 24]. For patients experiencing compression of the cervical spinal cord, antibiotic therapy and surgical decompression are the primary modes of therapy. After surgery, all patients showed significant improvements in VAS, JOA, and NDI scores after three months. Spinal decompression and reconstruction of cervical stability significantly contribute to improved spinal cord function and pain relief. The surgery typically involves removing a fraction of the anterior longitudinal ligament and affected intervertebral discs, debridement of spinal infection, epidural abscess drainage, and reconstruction. Anterior cervical fusion is accomplished through an autologous iliac strut bone graft or PEEK cage insertion. The anterior cervical approach can simultaneously remove infected tissue, drain abscesses, and reinstate the stability of the cervical spine. Moreover, the antibiotic therapy duration in surgical patients is typically shorter than that in conservative patients.

### Limitations

The present study has several limitations. First, cervical spinal brucellosis is extremely rare, and some cases detected early without symptoms of spinal cord compression can receive nonoperative treatment. The present study is a small sample, single-center study. Further large sample, multicenter studies are necessary to validate its clinical characteristic and outcomes of cervical spinal brucellosis. Second, the follow-up period of this study was 12–27 (17.9 ± 5.2) months, and there were no long-term outcomes available. As a result of the short follow-up period, the long-term efficacy of anterior cervical debridement, decompression, and fusion for cervical spinal brucellosis is presently unknown. Third, the surgery is performed by multiple surgeons and may result in variable individual patient treatment despite performing the same surgical procedure, including surgical techniques that may affect the success of treatment.

## Conclusions

Spinal brucellosis rarely affects the cervical region, but its impact is more dangerous due to potential complications such as paraplegia or tetraplegia arising from epidural abscesses that compress the spinal cord. Surgical debridement, along with essential antimicrobial therapy, is an effective strategy and can lead to satisfactory prognosis in managing cervical spinal brucellosis. Nonetheless, substantial large-sample, multicenter, and long-term follow-up prospective studies are necessary to demonstrate the clinical characteristic and validate the efficacy of surgical debridement along with essential antimicrobial therapy for cervical spinal brucellosis.

## Data Availability

No datasets were generated or analysed during the current study.

## Abbreviations

VAS: Visual analog scale
JOA: Japanese Orthopedic Association
NDI: Neck disability index
ESR: Erythrocyte sedimentation rate
CRP: C-reactive protein
STAT: Standard tube agglutination test
RBPT: Rose Bengal plate agglutination test
CT: Computed tomography
MRI: Magnetic resonance imaging

## References

[1] Esmaeilnejad-Ganji SM, Esmaeilnejad-Ganji SMR (2019). Osteoarticular manifestations of human brucellosis: a review. World J Orthop.

[2] Jiang W, Chen J, Li Q (2019). Epidemiological characteristics, clinical manifestations and laboratory findings in 850 patients with brucellosis in Heilongjiang Province, China. BMC Infect Dis.

[3] Gao M, Sun J, Jiang Z (2017). Comparison of tuberculous and brucellar spondylitis on magnetic resonance images. Spine.

[4] Tali ET, Koc AM, Oner AY (2015). Spinal brucellosis. Neuroimaging Clin N Am.

[5] Ma H, Zhang N, Liu J (2022). Pathological features of Brucella spondylitis: a single-center study. Ann Diagn Pathol.

[6] Liang C, Wei W, Liang X, De E, Zheng B (2019). Spinal brucellosis in Hulunbuir, China, 2011–2016. Infect drug Resist.

[7] Zhang Y, Zhang Q, Zhao CS (2019). Cervical brucellar spondylitis causing incomplete limb paralysis. Rev Soc Bras Med Trop.

[8] Baghi MAM, Al-Aani FK, Rahil A, Ayari B (2021). Brucellar cervical epidural abscess - A rare cause of neck pain. IDCases.

[9] Tao Z, Hua L, Chengwei Y, Bo F, Tao Q, Songkai L (2020). Three cases of Brucellar Spondylitis with noncontiguous multifocal involvement. World Neurosurg.

[10] Zhao G, Wang J, Xiang G, Zhou K, Nan W, Zhang H (2020). Cervical spinal tuberculosis combined with brucellosis. J Infect Dev Ctries.

[11] Khan MM, Babu RA, Iqbal J, Batas SN, Raza A (2020). Cervical epidural abscess due to Brucella Treated with decompression and instrumentation: a Case Report and Review of Literature. Asian J Neurosurg.

[12] Abulizi Y, Cai X, Xu T et al. Diagnosis and Surgical Treatment of Human Brucellar Spondylodiscitis. J Vis Exp. 2021;(171). 10.3791/61840 34096906

[13] Kaptan F, Gulduren HM, Sarsilmaz A (2012). Brucellar spondylodiscitis: comparison of patients with and without abscesses. Rheumatol Int.

[14] de Divitiis O, Elefante A (2012). Cervical spinal brucellosis: a diagnostic and surgical challenge. World Neurosurg.

[15] Zheng R, Xie S, Lu X (2018). A systematic review and Meta-analysis of epidemiology and clinical manifestations of human brucellosis in China. Biomed Res Int.

[16] Ulu-Kilic A, Karakas A, Erdem H (2014). Update on treatment options for spinal brucellosis. Clin Microbiol Infect.

[17] Erdem H, Elaldi N, Batirel A (2015). Comparison of brucellar and tuberculous spondylodiscitis patients: results of the multicenter Backbone-1 study. Spine Journal: Official J North Am Spine Soc.

[18] Alyousef M, Aldoghaither R (2018). First case of cervical epidural abscess caused by brucellosis in Saudi Arabia: a case report and literature review. IDCases.

[19] Resorlu H, Sacar S, Inceer BS (2016). Cervical spondylitis and epidural abscess caused by brucellosis: a Case Report and Literature Review. Folia Med (Plovdiv).

[20] Basavaraj A, Kulkarni R (2014). Cervical spine brucellosis presenting as fever with neck stiffness and cervical compressive myelopathy: a case report. Neurol India.

[21] Bodur H, Erbay A, Colpan A, Akinci E (2004). Brucellar spondylitis. Rheumatol Int.

[22] Koubaa M, Maaloul I, Marrakchi C (2014). Spinal brucellosis in South of Tunisia: review of 32 cases. Spine Journal: Official J North Am Spine Soc.

[23] Pappas G, Akritidis N, Bosilkovski M, Tsianos E, Brucellosis (2005). N Engl J Med.

[24] Unuvar GK, Kilic AU, Doganay M (2019). Current therapeutic strategy in osteoarticular brucellosis. North Clin Istanbul.

[25] Alp E, Doganay M (2008). Current therapeutic strategy in spinal brucellosis. Int J Infect Diseases: IJID : Official Publication Int Soc Infect Dis.

